# mSphere of Influence: High-throughput screens to rapidly assign function to microbial genes

**DOI:** 10.1128/msphere.00800-24

**Published:** 2025-01-29

**Authors:** Lori B. Huberman

**Affiliations:** 1 Plant Pathology and Plant-Microbe Biology Section, School of Integrative Plant Science, Cornell University, Ithaca, New York, USA; University of Georgia, Athens, Georgia, USA

**Keywords:** functional genomics, filamentous fungi, high-throughput screens

## Abstract

Lori Huberman works in the field of fungal genetics, with an emphasis on investigating the genetic mechanisms fungi use to sense and respond to the nutrients and toxins in their environment. In this mSphere of Influence article, she reflects on how “Rapid quantification of mutant fitness in diverse bacteria by sequencing randomly bar-coded transposons” by K. M. Wetmore, M. N. Price, R. J. Waters, J. S. Lamson, et al. (mBio 6:e00306-15, 2015, https://doi.org/10.1128/mBio.00306-15) made an impact on her by establishing technologies that open realistic possibilities for developing high-throughput screening methods to correlate phenotype to genotype in diverse fungal species.

## COMMENTARY

I enjoyed my biology classes, but it was not until I performed my first genetic screen as an undergraduate researcher that I knew biological research was what I wanted to do as a career. I worked in *Saccharomyces cerevisiae* as a graduate student and got used to a world where the functions of most of the genes in the genome were not only known but well studied by a community of many labs over decades. Moving into filamentous fungal research, where the majority of genes are uncharacterized, was a dream for this genetic screen lover. However, the sheer number of genes of unknown function in filamentous fungal genomes and the relatively small research community studying them begs the question of how we will truly come to understand these fascinating organisms at a molecular level.

Classical screening methods are extremely powerful but identifying mutants and determining the causative gene can be laborious and slow. With the advent of fast and relatively inexpensive genome sequencing, it is clear that vast numbers of genes and gene families of unknown function exist. Rapid identification of gene function through high-throughput screening methods is necessary to identify functions for the thousands of uncharacterized genes in microbial genomes. The majority of high-throughput screening methods available are designed with one of two types of microbes in mind: those with small enough genomes that performing mutagenesis and deep sequencing for each screen is possible or organisms with a large enough research community that genome-wide targeted mutant libraries can be constructed. Unfortunately, the majority of filamentous fungi fall into neither of these categories. In 2015, Wetmore et al. (1) designed an alternative to these methods in “Rapid quantification of mutant fitness in diverse bacteria by sequencing randomly bar-coded transposons”: random barcode transposon sequencing. In this study, Wetmore et al. (1) integrated transposons tagged with random, unique DNA barcodes into five bacterial species. They then sequenced the libraries and associated each barcode with its genomic location. Unlike standard transposon sequencing, the random barcodes make it possible to construct and sequence the library once and then use that library to rapidly perform any number of experiments merely by quantitatively sequencing the barcodes. This quantitative barcode sequencing determines the relative abundance of each barcode and, thus, each strain in the population. By comparing the change in the relative abundance of each strain in the starting population versus the final population, it is possible to determine the fitness of each mutant in the library. Wetmore et al. (1) and a follow-up study, “Mutant phenotypes for thousands of bacterial genes of unknown function” by Price et al. (2), in which they make and screen libraries in 32 bacterial species, demonstrate the power of this library construction method. Wetmore et al. (1) and Price et al. (2) performed hundreds of fitness experiments and associated mutant phenotypes with thousands of genes of unknown function, a mind-boggling number of screens and assigned phenotypes.

In contrast to many bacteria, filamentous fungi have notoriously low transformation efficiencies and genomes that range in size from 10s to 100s of megabases. Additionally, genome defense mechanisms present in filamentous fungi can make techniques like transposon mutagenesis, a common technique to increase insertion frequency in bacteria and some yeast, difficult or impossible. Constructing a new insertional mutagenesis library for every screen is costly in both researcher time and the sequencing necessary to map the insertional mutants. Randomly barcoded insertional mutagenesis enables the construction and sequencing of a single library that can be used to perform hundreds of experiments, opening the possibility of efficient high-throughput screening for filamentous fungi. It has already been successfully utilized in a non-model yeast (3).

Much of the functional genomics work in filamentous fungi has relied on techniques like transcriptomics and proteomics, which, while powerful, are not as efficient at gene characterization as screens specifically designed to identify phenotypes for fungal genes. Fungal genomes have thousands of uncharacterized genes that do not even have homologs with a known function, and the Joint Genome Institute has identified hundreds of fungal gene families of unknown function (4, 5). We are now working to generate randomly barcoded insertional mutagenesis libraries in filamentous fungi, which we hope will be instrumental in assigning functions to many of these uncharacterized genes and gene families. Going forward, combining random barcodes with tools like CRISPR activation and CRISPR interference could improve the ability to screen essential genes and investigate the effects of gene overexpression in high-throughput screens (6–8).

It is easiest to perform assays for changes in growth speed using insertional mutagenesis libraries, and this is how Wetmore et al. (1) and Price et al. (2) used the libraries they generated. My lab currently uses changes in the growth speed of mutants in randomly barcoded insertional mutagenesis libraries to identify genetic mechanisms fungi use to sense and respond to nutrients and other chemicals in their environment. Going forward, we will design experiments utilizing libraries like these to screen for additional phenotypes. Other groups have screened for changes in cell buoyancy and lipid content (3). It is intriguing to think about screening for additional cellular attributes like cell size or shape or combining these insertional mutant libraries with fluorescent dyes or other markers to screen for changes in cellular composition or organelles. For a biologist who started her research journey doing classical ethyl methanesulfonate mutagenesis screens, it is exciting to imagine the rapid pace of gene function discovery possible with high-throughput screening, expanding our knowledge of fungal genome content to encompass the functions those genomes encode.

## References

[1] Wetmore KM, Price MN, Waters RJ, Lamson JS, He J, Hoover CA, Blow MJ, Bristow J, Butland G, Arkin AP, Deutschbauer A. 2015. Rapid quantification of mutant fitness in diverse bacteria by sequencing randomly bar-coded transposons. mBio 6:e00306-15. doi:10.1128/mBio.00306-15 25968644 PMC4436071

[2] Price MN, Wetmore KM, Waters RJ, Callaghan M, Ray J, Liu H, Kuehl JV, Melnyk RA, Lamson JS, Suh Y, Carlson HK, Esquivel Z, Sadeeshkumar H, Chakraborty R, Zane GM, Rubin BE, Wall JD, Visel A, Bristow J, Blow MJ, Arkin AP, Deutschbauer AM. 2018. Mutant phenotypes for thousands of bacterial genes of unknown function. Nature 557:503–509. doi:10.1038/s41586-018-0124-0 29769716

[3] Coradetti ST, Pinel D, Geiselman GM, Ito M, Mondo SJ, Reilly MC, Cheng Y-F, Bauer S, Grigoriev IV, Gladden JM, Simmons BA, Brem RB, Arkin AP, Skerker JM. 2018. Functional genomics of lipid metabolism in the oleaginous yeast Rhodosporidium toruloides. Elife 7:e32110. doi:10.7554/eLife.32110 29521624 PMC5922974

[4] Grigoriev IV, Cullen D, Goodwin SB, Hibbett D, Jeffries TW, Kubicek CP, Kuske C, Magnuson JK, Martin F, Spatafora JW, Tsang A, Baker SE. 2011. Fueling the future with fungal genomics. Mycology 2:192–209. doi:10.1080/21501203.2011.584577

[5] Grigoriev IV, Nikitin R, Haridas S, Kuo A, Ohm R, Otillar R, Riley R, Salamov A, Zhao X, Korzeniewski F, Smirnova T, Nordberg H, Dubchak I, Shabalov I. 2014. MycoCosm portal: gearing up for 1000 fungal genomes. Nucleic Acids Res 42:D699–704. doi:10.1093/nar/gkt1183 24297253 PMC3965089

[6] Qi LS, Larson MH, Gilbert LA, Doudna JA, Weissman JS, Arkin AP, Lim WA. 2013. Repurposing CRISPR as an RNA-guided platform for sequence-specific control of gene expression. Cell 152:1173–1183. doi:10.1016/j.cell.2013.02.022 23452860 PMC3664290

[7] Gilbert LA, Larson MH, Morsut L, Liu Z, Brar GA, Torres SE, Stern-Ginossar N, Brandman O, Whitehead EH, Doudna JA, Lim WA, Weissman JS, Qi LS. 2013. CRISPR-mediated modular RNA-guided regulation of transcription in eukaryotes. Cell 154:442–451. doi:10.1016/j.cell.2013.06.044 23849981 PMC3770145

[8] Bikard D, Jiang W, Samai P, Hochschild A, Zhang F, Marraffini LA. 2013. Programmable repression and activation of bacterial gene expression using an engineered CRISPR-Cas system. Nucleic Acids Res 41:7429–7437. doi:10.1093/nar/gkt520 23761437 PMC3753641

